# Correction: Increased Iron Sequestration in Alveolar Macrophages in Chronic Obtructive Pulmonary Disease

**DOI:** 10.1371/journal.pone.0104601

**Published:** 2014-07-30

**Authors:** The PLOS ONE Staff

The word “Obstructive” is misspelled in the article title. The correct title is: Increased Iron Sequestration in Alveolar Macrophages in Chronic Obstructive Pulmonary Disease. The correct citation is: Philippot Q, Deslée G, Adair-Kirk TL, Woods JC, Byers D, et al. (2014) Increased Iron Sequestration in Alveolar Macrophages in Chronic Obstructive Pulmonary Disease. PLoS ONE 9(5): e96285. doi:10.1371/journal.pone.0096285
